# Congenital Cytomegalovirus Pneumonitis Mimicking Childhood Interstitial Lung Disease: A Case Report and Review of Diagnostic Challenges

**DOI:** 10.7759/cureus.87869

**Published:** 2025-07-13

**Authors:** Khalfan Al Abdali, Talal Al Wardi, Ruba Elfatih, Yarab Al Bulushi, Majid Al Jabri

**Affiliations:** 1 Department of Pediatrics, Nizwa Hospital, Ministry of Health, Nizwa, OMN; 2 Department of Radiology, College of Medicine and Health Sciences, Sultan Qaboos University Hospital, Muscat, OMN; 3 Department of Child Health, College of Medicine and Health Sciences, Sultan Qaboos University Hospital, Muscat, OMN

**Keywords:** childhood interstitial lung disease, congenital cytomegalovirus infection, fiberoptic flexible bronchoscopy, neonatal respiratory distress, whole-exome sequencing

## Abstract

We present a case of a full-term neonate with congenital cytomegalovirus (CMV) infection manifesting as severe pneumonitis. The patient exhibited intrauterine growth restriction (IUGR), early onset of respiratory distress, and persistent oxygen dependency. High viral load in urine and plasma supported the diagnosis, confirmed within the first three weeks of life. Despite treatment with oral valganciclovir for six months, persistent tachypnea, failure to thrive, and radiological findings prompted a broader differential diagnosis, including childhood interstitial lung disease (chILD). Bronchoscopy and whole-exome sequencing (WES) were performed. This case underscores the need for multidisciplinary evaluation in neonates with unexplained or protracted respiratory illness and highlights the potential overlap between congenital CMV pneumonitis and genetic interstitial lung disease (ILD).

## Introduction

Congenital cytomegalovirus (CMV) is the most common congenital viral infection worldwide, affecting approximately 0.5-1% of all live births. While most cases are asymptomatic, symptomatic infants may present with a broad range of clinical features, including hepatosplenomegaly, thrombocytopenia, neurodevelopmental delay, sensorineural hearing loss (SNHL), and intrauterine growth restriction (IUGR). Pulmonary involvement, including pneumonitis, is rare but has been described in both congenital and perinatal CMV infections [1,2].

In this report, we present a case of congenital CMV pneumonitis in a term neonate with severe respiratory distress and persistent hypoxemia. The diagnostic pathway included virological confirmation, radiological imaging, and extensive evaluation for differential diagnoses such as childhood interstitial lung disease (chILD). This case highlights the diagnostic challenges of congenital CMV pneumonitis and the risk of long-term lung complications. It underscores the need for early recognition, timely antiviral therapy, and further evaluation in persistent cases, as well as long-term follow-up to monitor for growth, hearing, and respiratory outcomes.

## Case presentation

A full-term female neonate was born via cesarean section due to oligohydramnios. Birth weight was 2.1 kg (less than the third percentile), head circumference 30cm (less than the third percentile), with symmetrical IUGR. Appearance, Pulse, Grimace, Activity, and Respiration (APGAR) scores were 8 and 9 at one and five minutes, respectively. The mother had symptoms of viral illness, including cough and fever, before the date of delivery.

The neonate developed respiratory distress within the first two hours of life and required mechanical ventilation. Echocardiography confirmed persistent pulmonary hypertension of the newborn (PPHN), and sildenafil was initiated as inhaled nitric oxide (iNO) was not available in the hospital. After extubation on day 7 of life, the infant remained dependent on oxygen. The sepsis workup was negative, and antibiotics were discontinued. Initial labs revealed a white blood cell (WBC) count of 29.7 × 10^9^/L, neutrophils 20.6 × 10^9^/L, platelet count 191 × 10^9^/L, hemoglobin (Hb) 15 g/dL, alanine aminotransferase (ALT) 51 U/L, aspartate aminotransferase (AST) 63 U/L, and C-reactive protein (CRP) less than 1 mg/L, as shown in Tables 1-2.

**Table 1:** Initial complete blood count (CBC) findings at the time of presentation

Test Name	Value	Unit	Reference Range
Hemoglobin (Hb) in blood	15	g/dL	12.5-19.5
Hematocrit in blood	45	%	44-62
Red blood cells in blood	5.02	10^12^/L	4.4-6.8
Mean cell volume	89.8	fL	77-95
Mean cell Hb	30	pg	31-37
Mean cell Hb concentration	33.4	g/dL	30-36
RBC distribution width	15.2	%	11.5-16.5
Platelet count in blood	191	10^9^/L	150-450
Mean platelet volume in blood	10.4	fL	9.0-12.5
White blood cells in blood	29.7	10^9^/L	5-12
Neutrophils (absolute) in blood	20.6	10^9^/L	2.0-7.5
Lymphocytes (absolute) in blood	4.19	10^9^/L	2.0-11.5
Monocytes (absolute) in blood	2.14	10^9^/L	0.2-1.6

**Table 2:** Initial liver function test (LFT) values at the time of presentation

Test Name	Value	Unit	Reference Range
Bilirubin total in serum/plasma	32.9	µmol/L	3.42-23.86
Alanine transaminase (ALT)	51	U/L	5-40
Aspartate aminotransferase (AST)	63	U/L	5-40
Total protein in serum/plasma	42	g/L	60-80
Alkaline phosphatase in serum/plasma	123	U/L	90-273
Albumin in serum/plasma	29	g/L	33-45

**Figure 1:**
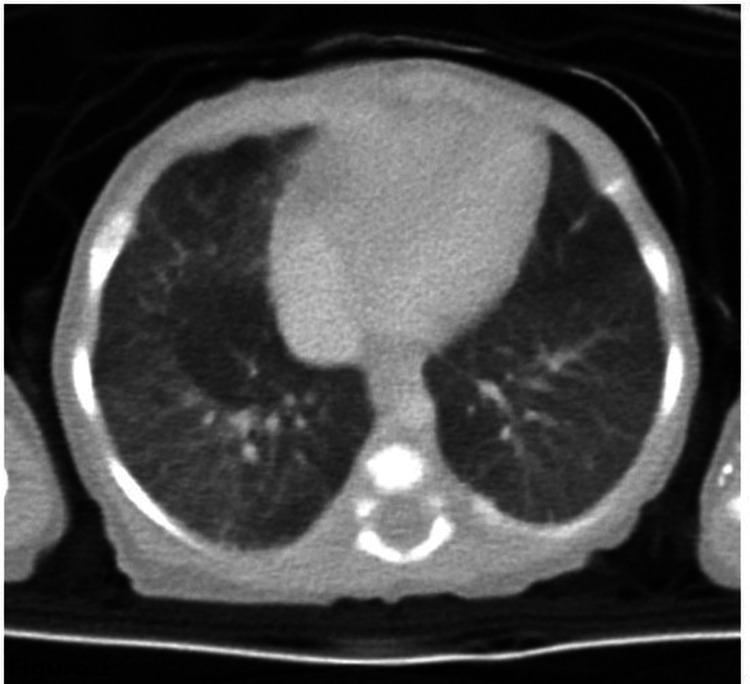
Axial high-resolution CT image at 27 days of life The image demonstrates diffuse mosaic attenuation in both lungs, possibly attributable to small airway disease.

High-resolution chest CT showed diffuse mosaic attenuation, suggestive of small airway disease (Figure 1).

A 10-day course of dexamethasone (DART protocol) was administered for severe respiratory distress before confirmation of CMV infection. TORCH (*Toxoplasma gondii*, rubella, CMV, and herpes simplex virus (HSV)) screening revealed positive CMV immunoglobulin G (IgG) and immunoglobulin M (IgM) antibodies. Urine CMV polymerase chain reaction (PCR) testing showed 1,937,547 copies/mL, while plasma CMV PCR revealed 5,596 copies/mL. Although congenital CMV infection was confirmed within the first three weeks of life through PCR testing of urine and plasma, initiation of valganciclovir (16 mg/kg BID) was delayed until six weeks of age and continued for six months. This delay was due to initial diagnostic uncertainty related to overlapping bacterial pneumonia and PPHN, which required stabilization and multidisciplinary evaluation. Additionally, administrative processes for medication approval and comprehensive parental counseling contributed to the later start of antiviral therapy.

**Figure 2:**
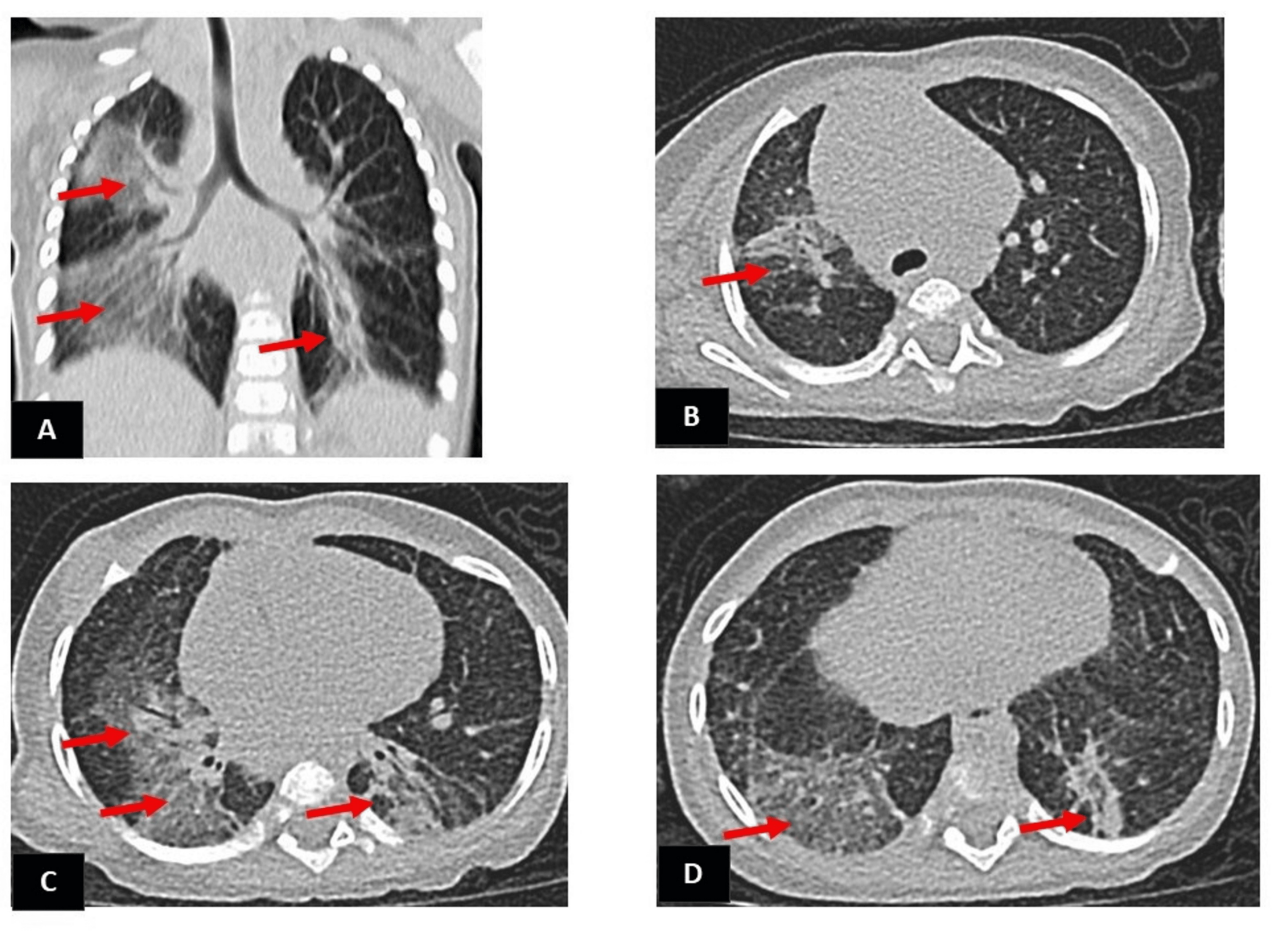
High-resolution CT chest performed at six months of age (A) Coronal oblique image (red arrows) shows bilateral patchy ground-glass densities containing plate atelectasis involving the upper, middle, and lower lobes on the right side in addition to the left lower lobe. (B) The image demonstrates an ill-defined ground-glass opacity in the anterior segment of the right upper lobe (red arrow). (C) Patchy ground-glass opacities are seen in the medial segment of the right middle lobe and the superior segments of both lower lobes (red arrows). (D) The image shows an ill-defined ground-glass opacity in the superior segment of the right lower lobe and plate atelectasis in the superior segment of the left lower lobe (red arrows).

As part of a multidisciplinary evaluation for infants presenting with severe respiratory distress and ongoing oxygen requirements, this patient was closely followed by pediatric pulmonologists at Sultan Qaboos University Hospital (SQUH). A high-resolution chest CT was repeated at six months of age to evaluate for residual pulmonary sequelae and to explore alternative or coexisting diagnoses. It was reviewed with a pediatric radiologist to better characterize the findings. The imaging demonstrated mosaic attenuation, patchy ground-glass opacities, and mild interlobular septal thickening, features that can be seen in post-infectious sequelae but also overlap with patterns described in chILD (Figure 2).

While certain features were nonspecific, the clinical course, marked by gradual improvement and oxygen weaning, favored a diagnosis of post-viral changes rather than primary chILD.

Although valganciclovir (16 mg/kg BID) had been initiated at six weeks of age and completed over six months, the infant continued to exhibit baseline tachypnea, digital clubbing (more prominent in the toes, and failure to thrive (weight 6 kg at seven months, less than third percentile)), despite adequate caloric intake. These persistent clinical features and atypical imaging findings prompted referral for comprehensive evaluation.

**Figure 3:**
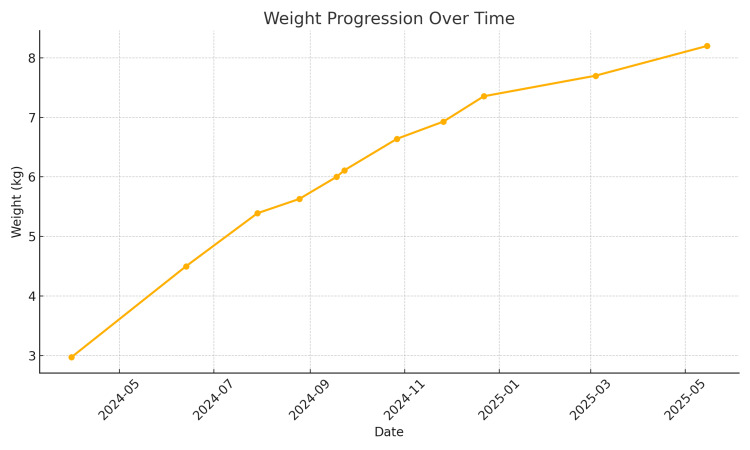
Longitudinal follow-up of weight gain

Fiberoptic flexible bronchoscopy was performed at eight months of age to assess airway anatomy and collect bronchoalveolar lavage (BAL) samples. BAL was negative for bacterial, mycobacterial, fungal, and viral pathogens. Simultaneously, whole-exome sequencing (WES) targeting pulmonary interstitial lung disease (ILD) genes was conducted and returned negative for known pathogenic variants. Over the following months, the infant was successfully weaned from oxygen, with gradual improvement in weight gain (8.2 kg by 15 months), and continued normal developmental progress (Figure 3).

## Discussion

CMV infection presents with a wide spectrum of clinical features, which may be apparent at birth or emerge later in infancy. Common manifestations include jaundice, petechiae secondary to thrombocytopenia, symmetrical IUGR, hepatosplenomegaly, microcephaly, intracerebral calcifications, chorioretinitis, SNHL, and neurodevelopmental delay [3].

Pulmonary involvement in congenital CMV is rare, with pneumonitis reported in less than 1% of symptomatic cases [4]. In our case, the presence of early respiratory distress, need for supplemental oxygen, and markedly elevated viral load raised clinical suspicion for CMV pneumonitis.

Accurate diagnosis of CMV pneumonitis in infants is often complex due to its overlap with other neonatal and ILDs. The detection of CMV DNA within the first three weeks of life, typically from urine, is critical to confirm congenital rather than postnatally acquired infection [5]. Although histopathological identification of CMV inclusion bodies in lung tissue remains the diagnostic gold standard, lung biopsy is seldom performed in neonates due to its invasive nature [6]. When obtained, pathological findings may reveal diffuse alveolar damage, swollen pneumocytes, alveolar wall edema, interstitial inflammation, or fibrosis [6].

In clinical settings, diagnosis is generally established through a combination of clinical presentation, radiologic imaging, and virological assays. PCR testing for CMV DNA in urine, blood, or BAL fluid remains a cornerstone of laboratory confirmation [5]. Radiologically, high-resolution CT may reveal ground-glass opacities, interstitial infiltrates, or small pulmonary nodules. However, these findings are nonspecific and may resemble other neonatal ILDs such as those classified under chILD [7].

Persistent signs such as tachypnea, digital clubbing, and inadequate weight gain, despite antiviral therapy, should prompt further evaluation for alternative or coexisting diagnoses. In such contexts, bronchoscopy with BAL and genetic testing (e.g., WES targeting surfactant protein mutations) are essential components of a thorough diagnostic workup [8,9].

Although CMV pneumonitis typically induces an inflammatory process, chronic sequelae, including parenchymal fibrosis and irreversible lung damage, may occur. Antiviral therapy, particularly when initiated during the neonatal period, is associated with better outcomes and may attenuate the risk of long-term complications [10]. Valganciclovir is currently recommended for symptomatic infants, especially those with central nervous system or pulmonary involvement, and early initiation appears to limit tissue injury and fibrotic remodeling [10].

This case underscores the diagnostic challenge in distinguishing post-infectious pulmonary changes from primary ILD in infants. The absence of ongoing infection or genetic abnormalities, along with clinical improvement over time, supported a diagnosis of post-viral sequelae. The role of a multidisciplinary team, comprising pediatric pulmonologists, infectious disease specialists, and radiologists, was essential in guiding diagnosis, excluding other etiologies, and planning long-term follow-up.

This highlights the importance of specialist radiologic input when evaluating complex neonatal lung disease, as certain imaging features may overlap with chILD, necessitating careful multidisciplinary interpretation to guide management and prognosis.

## Conclusions

Congenital CMV infection should be considered in neonates presenting with unexplained respiratory distress, particularly in the context of IUGR and high viral load. Persistent respiratory symptoms and radiographic abnormalities following antiviral therapy necessitate further investigation to rule out underlying ILD. This case reinforces the value of early diagnosis, timely antiviral treatment, and a multidisciplinary approach to optimize clinical outcomes and monitor for potential long-term pulmonary complications.
